# DAILY STRESSORS, POSITIVE EXPERIENCES, AND DAILY FATIGUE AMONG DEMENTIA FAMILY CAREGIVERS

**DOI:** 10.1093/geroni/igae098.2268

**Published:** 2024-12-31

**Authors:** Eunju Lee, Kyungmin Kim, Yin Liu, Steven Zarit

**Affiliations:** Seoul National University, Seoul, Republic of Korea; Seoul National University, Seoul, Republic of Korea; Utah State University, Logan, Utah, United States; The Pennsylvania State University, University Park, Pennsylvania, United States

## Abstract

Family caregivers of individuals with dementia (IWD) suffer from physical and mental fatigue, but little is known about daily fluctuation of caregiver fatigue in response to daily care-related and non-care experiences. Caregivers are also likely to encounter conflict with other family members on caregiving issues—one of the secondary stressors that may amplify their fatigue. This study examined the within-person associations of daily fatigue with care-related stressors (i.e., relative’s behavioral and psychological symptoms of dementia), non-care stressors, and positive experiences, and whether family conflict on caregiving issues exacerbates the relationship between daily experiences and fatigue. We analyzed a sample of 173 family caregivers of IWD who participated in daily interviews across 8 consecutive days (N = 1,359). Participants reported daily fatigue and daily experiences each day, while family conflict was reported at baseline. Multilevel models showed that experiencing more care-related and non-care stressors than usual was associated with higher levels of daily fatigue, whereas having more positive experiences than usual was associated with lower levels of fatigue. At the between-person level, caregivers with more care-related stressors on average were more likely to show higher levels of fatigue. Further, family conflict on caregiving issues exacerbated the relationship between daily care-related stressors and fatigue. The findings suggested that not only care-related stressors, but also non-care daily experiences were related to fluctuations of daily fatigue among family caregivers. Exposure to positive experiences and resolving family disagreement concerning caregiving issues may help caregivers relieve fatigue and tiredness.

